# Metastasis-Free Survival in Patients with Biochemical Recurrence After Robot-Assisted Radical Prostatectomy: A Multicenter, Retrospective Cohort Study in Japan (MSUG94 Group)

**DOI:** 10.3390/curroncol33010056

**Published:** 2026-01-18

**Authors:** Minori Nezasa, Masayuki Tomioka, Tomoyuki Tatenuma, Takeshi Sasaki, Yoshinori Ikehata, Akinori Nakayama, Masahiro Toide, Tatsuaki Yoneda, Kazushige Sakaguchi, Kazuhide Makiyama, Takahiro Inoue, Hiroshi Kitamura, Kazutaka Saito, Fumitaka Koga, Shinji Urakami, Takuya Koie

**Affiliations:** 1 Department of Urology, Gifu University Graduate School of Medicine, Gifu 501-1194, Japan; nezasa.minori.e7@f.gifu-u.ac.jp (M.N.); tomioka.masayuki.e4@f.gifu-u.ac.jp (M.T.); 2 Department of Urology, Yokohama City University, Yokohama 236-0004, Japan; tatenuma@yokohama-cu.ac.jp (T.T.); makiya@yokohama-cu.ac.jp (K.M.); 3 Department of Nephro-Urologic Surgery and Andrology, Mie University Graduate School of Medicine, Tsu 514-8507, Japan; t-sasaki@med.mie-u.ac.jp (T.S.); tinoue28@clin.medic.mie-u.ac.jp (T.I.); 4 Department of Urology, University of Toyama, Toyama 930-0194, Japan; ikehatay@med.u-toyama.ac.jp (Y.I.); hkitamur@med.u-toyama.ac.jp (H.K.); 5 Department of Urology, Dokkyo Medical University Saitama Medical Center, Koshigaya 343-8555, Japan; akinori@dokkyomed.ac.jp (A.N.); kzsaito@dokkyomed.ac.jp (K.S.); 6 Department of Urology, Tokyo Metropolitan Cancer and Infectious Diseases Center Komagome Hospital, Tokyo 113-8677, Japan; masahiro_toide@tmhp.jp (M.T.); f-koga@cick.jp (F.K.); 7 Department of Urology, Seirei Hamamatsu General Hospital, Hamamatsu 430-8558, Japan; yonet@sis.seirei.or.jp (T.Y.); shinji.urakami@toranomon.gr.jp (S.U.); 8 Department of Urology, Toranomon Hospital, Tokyo 105-8470, Japan; sakaguchi-k@toranomon.gr.jp

**Keywords:** prostate cancer, robot-assisted radical prostatectomy, biochemical recurrence, metastasis-free survival, lymphovascular invasion

## Abstract

Robot-assisted radical prostatectomy (RARP) is the definitive surgical treatment for localized prostate cancer (PCa). Some patients with post-RARP biological recurrence (BCR) eventually develop distant metastases and subsequent PCa-related mortality. The objective of this study was to clarify the predictive factors for the risk of metastatic disease after BCR in patients with PCa who underwent RARP. This multicenter retrospective cohort study was conducted in nine Japanese institutions and enrolled 491 men with BCR, detected between 2011 and 2024. During the median 59-month follow-up period, 44 patients (9.0%) had radiological confirmation of distant metastasis. Patients with developed metastases after BCR exhibited higher biopsy Gleason grade and pathological T stage, increased lymphovascular invasion (LVI) in the surgical specimen, and a shorter interval from RARP to BCR. In univariate analysis, LVI and a time to BCR after RARP of ≤14.9 months were significant predictors of distant metastasis. In the multivariate analysis, LVI constituted a significant independent predictor of distant metastasis (*p* = 0.011). The 3-year metastasis-free survival (MFS) rates were 85.5% and 94.1% in patients with and without LVI, respectively. The MFS was significantly prolonged in patients with negative LVI compared to those with positive LVI (*p* = 0.007). In Japanese males with BCR after RARP, LVI was identified as an independent predictor of metastatic progression.

## Simple Summary

Prostate cancer is among the most prevalent cancers in men, with surgical removal of the prostate being the standard treatment. While PSA has been used as a marker for the possibility of subsequent cancer recurrence following surgery, cases exhibiting PSA recurrence do not consistently develop distant metastases. In this study, 491 Japanese men who experienced PSA recurrence after undergoing robot-assisted radical prostatectomy were observed over a period of five years. The study found that 9% of patients experienced distant metastasis. Multivariate analysis revealed that positive lymphovascular invasion by prostate cancer cells in the surgical specimen is an independent predictor of postoperative distant metastasis.

## 1. Introduction

Radical prostatectomy (RP), particularly robot-assisted RP (RARP), has emerged as a prevalent surgical intervention for the definitive treatment of localized prostate cancer (PCa) [1,2,3]. In numerous long-term studies of patients who underwent RP, the 10-year BCR-free survival rate ranges from 73% to 77%, and cancer-specific survival (CSS) exceeds 90% [1,2]. Although significant long-term cancer control is achieved in many patients with PCa, some patients of biochemical recurrence (BCR) develop after surgery [3]. In long-term cohort studies, the median interval from the BCR occurrence to the progression of metastatic disease following RP was 8 years [2,4]. Moreover, the 15-year metastasis-free survival (MFS) and CSS rates in patients with BCR after RP were 75.8% and 83.6%, respectively [1]. Several studies have shown that RP prolongs the interval between BCR onset and PCa-related death, thereby increasing long-term risks of metastasis and cancer-related mortality.

In contrast, the clinical course and oncological outcomes after BCR demonstrate significant variability among individual cases. Specifically, patients with PCa may exhibit high-grade clinical features that are associated with early disease progression [1,2,3,5,6,7]. A high Gleason grade (GG), pathological T stage, prostate-specific antigen (PSA) doubling time (PSADT), and the interval from RP to BCR have been identified as independent predictors of PCa-specific mortality [1,5]. Given the prolonged overall survival (OS) of patients with PCa, MFS has been established as an alternative prognostic indicator, and its utility has been confirmed in many studies [6,7]. However, only a limited number of reports have focused on the identification of the predictive factors for the development of metastatic disease following BCR in patients with PCa who have undergone RARP, which is currently the most prevalent surgical treatment.

This multicenter retrospective study aimed to evaluate the MFS after BCR in patients with PCa who were treated with RARP, and to assess the clinicopathological factors that are associated with the development of metastasis.

## 2. Materials and Methods

### 2.1. Study Population and Preoperative Clinical Variables

This study was reviewed by the Gifu University Ethics Review Committee and received approval under number 2021-A050. The present study is of a retrospective nature; hence, the requirement for written informed consent was waived by implementing an opt-out procedure as per Japanese ethics committee regulations and ethical guidelines regarding the published results of retrospective and observational studies. Additional research details can be found at https://rinri.med.gifu-u.ac.jp/esct/publish_document.aspx?ID=2305 (accessed on 1 October 2025).

This multicenter, retrospective cohort study was carried out from September 2012 to January 2024 and enrolled 3473 patients with PCa who underwent RARP (MSUG94 cohort) at nine facilities in Japan. The clinical data collected for the study participants included age, height, weight, serum initial PSA level, GG on biopsy, clinical stage, risk stratification using the National Comprehensive Cancer Network risk stratification guidelines [8], Eastern Cooperative Oncology Group performance status, and administration of neoadjuvant or adjuvant therapy. This study did not collect data on whether the enrolled patients underwent magnetic resonance imaging evaluation before the prostate biopsy. The evaluation and classification of GGs using biopsy and prostatectomy specimens were conducted in accordance with the 2014 guidelines established by the International Society of Urologic Pathology (ISUP) [9]. The extent of pelvic lymph node dissection (PLND) was categorized as either limited, involving only the obturator lymph nodes, or extended, involving the common iliac vascular ureteric crossing, including or excluding the presacral lymph nodes [10,11]. The extent of PLND, the decision to perform nerve-sparing, and the inclusion of other related procedures were determined by an individual surgeon or institution. The percentage of positive biopsy cores was defined as the proportion of sampled cores that contained carcinomas.

### 2.2. Surgical and Pathological Variables

The following data were documented for each surgical outcome and pathological characteristic: console time, estimated blood loss, GG of surgical specimens, tumor (T) and node (N) stages of the surgical specimens, number of dissected lymph nodes, surgical margin status, and presence of lymphovascular invasion (LVI). Tumors were evaluated and staged in accordance with the American Joint Committee on Cancer 8th Edition “Cancer Staging Manual” [12]. All prostatectomy specimens were sectioned using the whole-mount staining method and assessed for PCa as recommended by the ISUP2014 guidelines [9]. Pathological assessment was conducted at each institution; however, a centralized pathology review was not performed.

### 2.3. Follow-Up Protocol

For all enrolled patients, serum PSA levels were monitored every 3 months after RARP. However, the testosterone levels were not examined in all patients. BCR was defined as a serum PSA level that exceeds 0.2 ng/mL, as indicated by at least two consecutive measurements. In the event that PSA levels failed to postoperatively decrease to <0.2 ng/mL, the date of RARP was taken as the date of BCR occurrence [13].

### 2.4. Endpoints and Statistical Analysis

The primary endpoint of this study was MFS, with MFS onset defined as the occurrence of BCR. Secondary endpoints were used to evaluate the incidence of metastatic lesions after BCR and examine the impact of clinicopathological prognostic factors on the possibility of metastatic disease. The presence of distant metastases was radiologically confirmed using computed tomography, magnetic resonance imaging, or bone scintigraphy. Imaging examinations were performed if clinical indications were present or during routine follow-up, in accordance with institutional policies.

R v4.4.1 (R Foundation for Statistical Computing, Vienna, Austria) was utilized for all the analyses. The Wilcoxon signed-rank test was employed to compare continuous variables, whereas the chi-square or Fisher’s exact test was used to compare categorical variables. MFS was estimated using the Kaplan–Meier method, and the log-rank test was used to assess the associations between metastasis and covariates. Multivariate analysis was performed using Cox proportional hazards analysis, and hazard ratios (HRs) with 95% confidence intervals (CIs) were calculated for the clinicopathological risk factors. Two-sided *p*-values were used in the analysis, with statistical significance defined as a *p*-value < 0.05.

## 3. Results

### 3.1. Patients and Characteristics

Of the 3473 patients with PCa who participated in the study, 2853 were excluded because they did not develop BCR, 12 were excluded for missing critical clinicopathological data, and 117 were excluded for having received neoadjuvant or adjuvant systemic therapy. Subsequently, 491 patients who experienced BCR after RARP were included in the analysis. During a median follow-up period of 59 (interquartile range [IQR], 42.5–73) months, 44 cases (9.0%) exhibited radiographic evidence of distant metastasis, and 7 cases (1.4%) died from PCa. Among these 44 patients, the median time from RARP to metastasis was 24 (IQR, 7–34) months, and the median time from BCR to metastasis was 6 (IQR, 1–19) months.

Table 1 summarizes the clinicopathological variables of the 491 patients who were included in the analysis. Patients were divided into two groups: those who did not develop metastasis (non-metastatic group) and those who eventually developed metastasis (metastatic group). The median age at surgery was found to be 68 (IQR, 64–72) years, and the median initial PSA level was recorded as 9.8 (IQR, 6.8–15.4) ng/mL. Overall, 44.2% of patients had a biopsy GG ≥ 4, and 58.5% had a pathological T stage ≥ 3. At the time of treatment initiation for BCR, the median PSA for the non-metastatic and metastatic groups were 0.25 (IQR, 0.22–0.31) ng/mL and 0.34 (IQR, 0.26–1.35) ng/mL, respectively. Among patients who received salvage radiotherapy, the median time from BCR to initiation of salvage radiotherapy was 1.8 months (IQR, 0.5–4.4 months) in the non-metastasis group and 2.8 months (IQR, 1.4–5.2 months) in the metastasis group.

**Figure 1:**
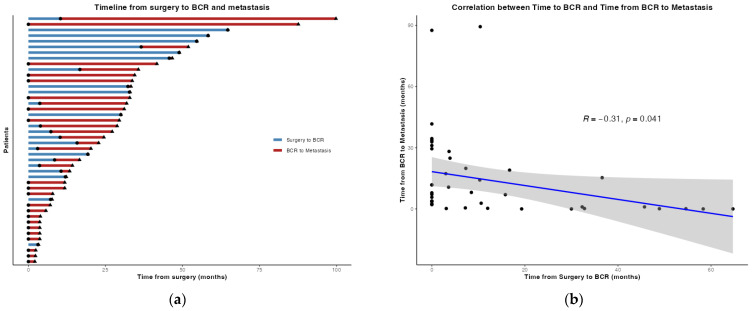
(**a**) The timeline from robot-assisted radical prostatectomy (RARP) to biochemical recurrence (BCR) and subsequent distant metastasis was examined among a group of patients who eventually developed metastasis. A solid circle (●) marks the timing of BCR, and a triangle (▲) marks the occurrence of distant metastasis; (**b**) a correlation was identified for the time from RARP to BCR and the interval from BCR to distant metastasis among patients with metastasis. Scatter plot showing individual patients (black dots) with a linear regression line (blue) and a 95% confidence interval (grey shading).

The clinical timelines for the 44 patients are presented in Figure 1a, and the relationship between the time to BCR and RARP and the time from BCR to metastasis is shown in Figure 1b.

### 3.2. Metastasis-Free Survival

**Figure 2:**
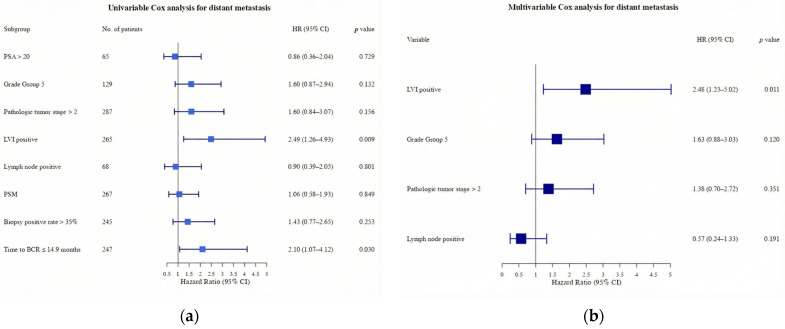
(**a**) Forest plot of univariable Cox regression analysis for time from biochemical recurrence to distant metastasis (*n* = 491); (**b**) multivariable Cox regression analysis for time from BCR to distant metastasis. HR, hazard ratio; CI, confidence interval; PSA, prostate-specific antigen; PSM, positive surgical margin.

The MFS rate for the entire cohort was 93.8% (95% CI, 91.6–96.0), 89.9% (95% CI, 86.9–93.0), and 89.3% (95% CI, 86.2–92.6) at 1, 3, and 5 years, respectively. In univariate analysis, the presence of LVI and a time to BCR after RARP of ≤14.9 months were significant predictors of distant metastasis (Figure 2a). In the multivariate Cox proportional hazards model, LVI was a significant independent predictor of distant metastasis (*p* = 0.011; Figure 2b).

**Figure 3:**
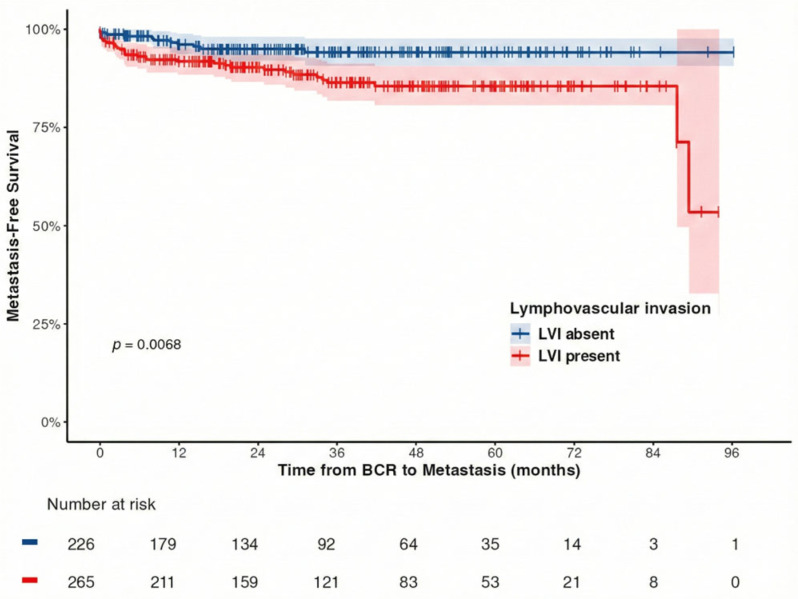
Kaplan–Meier estimates for metastasis-free survival (MFS) based on the presence or absence of lymphovascular invasion (LVI) in resected specimens following robot-assisted radical prostatectomy. The 3-year MFS rate was 86.4% in patients with positive LVI and 94.1% in those with negative LVI (*p* = 0.007).

Regarding MFS, patients were divided into two groups based on the presence or absence of LVI in the resected specimen. The 3-year and 5-year MFS rates were determined to be 86.4% (95% CI, 81.9–91.2) and 85.5% (95% CI, 80.7–90.6), respectively, in patients with positive LVI. In contrast, the 3-year and 5-year MFS rates in patients with negative LVI were found to be 94.1% (95% CI: 90.7–97.6) and 94.1% (95% CI: 90.7–97.6), respectively, and this indicated a significant prolongation of MFS in those with negative LVI compared to those with positive LVI (*p* = 0.007; Figure 3).

## 4. Discussion

Despite extensive research on the relationship between the time from RP to BCR and oncological outcomes, a consensus remains inconclusive. Numerous reports indicate that a shorter interval to BCR after RP is a significant risk factor for poor MFS and CSS [4,5,14,15,16,17]. However, other studies have shown no correlation between BCR and MFS [1,2,18]. Concurrent studies on RARP have indicated that the time to BCR is an independent predictor of metastasis, whereas time from metastasis to death is a predictor of OS [19]. Regarding these discrepancies, a hypothesis has been proposed that attributes these discrepancies to statistical adjustment bias [20]. Several studies have identified PSADT as a highly effective predictor of prognosis following BCR [1,2,5,17,18,21,22,23]. However, PSADT has been shown to exhibit a strong correlation with the time to BCR and progression to metastatic disease, suggesting that its strong association may obscure the prognostic contribution of other variables [20]. In the present cohort, a significant correlation was observed between the time to BCR after RARP and the time from BCR to metastasis. Moreover, univariate analysis indicated that patients who experienced early postoperative BCR exhibited significantly worse MFS outcomes.

Although various pathological factors are widely recognized as predictors of BCR, few studies have specifically investigated pathological predictors of distant metastasis. A recent meta-analysis of pathological factors demonstrated that seminal vesicle invasion, positive resection margins, prostatic capsule invasion, vascular invasion, LVI, and perineural invasion are useful predictors of BCR [24]. Multivariate analysis indicated that all of the aforementioned variables had a statistically significant impact on increasing BCR risk, with their prognostic impact being nearly equivalent (HR, 1.59–2.03) [24]. Conversely, the univariate analysis of our study, which utilized distant metastasis as the endpoint, identified the presence of LVI and a postoperative time to BCR ≤ 14.9 months as significant factors that increased the risk of metastasis (*p* = 0.009 and *p* = 0.030, respectively). The multivariate analysis identified LVI as a significant independent predictor of metastatic disease progression. However, it is noteworthy that only approximately 9% of this cohort developed metastases, and this underscores the need for cautious interpretation of the results. Additionally, lymph node involvement is widely accepted as a significant prognostic factor for both MFS and OS [25,26,27,28,29]. In studies that stratified prognosis based on the number of positive lymph nodes, the HR was determined to be 1.38. Patients with 1–2 positive lymph nodes exhibited a 10-year CSS rate of 70–80%, whereas those with ≥3 positive nodes demonstrated a significantly poorer outcome of 33% [30]. However, the findings of the present study suggest that lymph node metastasis is not an independent predictor of distant metastases. This discrepancy may be attributed to the comparatively brief follow-up duration, which contrasts with the extended follow-up periods in previous studies. Moreover, the relatively limited number of cases with distant metastasis may have prevented the detection of the prognostic impact of lymph node metastasis. Notably, the study population included a small proportion of patients with a low number of positive lymph nodes. These patients are typically considered as having a favorable long-term prognosis [30], which may have led to a relative reduction in the overall impact of lymph node metastasis. Patients with pathologically confirmed lymph node positivity frequently receive immediate adjuvant or early salvage therapy, including radiation therapy. Systemic targeted therapy delays metastasis in patients with a high probability of developing BCR [31]. Therefore, lymph node metastasis in this study may not have demonstrated a significant prognostic impact.

LVI has been consistently associated with adverse outcomes across multiple malignancies beyond prostate cancer, including bladder, colorectal, and non-small-cell lung cancers [32,33,34]. In node-negative bladder cancer treated with radical cystectomy, LVI has been reported to increase with advancing pathologic stage and to independently predict local and distant recurrence, as well as worse cause-specific and overall survival after adjustment for conventional clinicopathologic variables [32]. In resected non-small-cell lung cancer, LVI has likewise been associated with inferior overall survival regardless of tumor stage, while pleural invasion, N2 nodal disease, and age also contribute to risk stratification in multivariable models [33]. In the context of colorectal cancer, LVI is recognized as a highly malignant pathological feature that has been shown to adversely impact oncological outcomes. Furthermore, the presence of vascular invasion, especially extravascular invasion, and tumor budding have been demonstrated to serve as significant predictors of postoperative recurrence and cancer-related mortality [34,35]. LVI is widely regarded as a valuable prognostic factor in numerous malignancies. Nevertheless, other pathological characteristics, including vascular invasion, the invasive pattern and depth of the tumor, the pathological stage, and the histological tumor grade, as well as molecular biomarkers, have also been identified as predictors of unfavorable oncological outcomes. Therefore, further investigation is warranted regarding the utility of LVI as a prognostic factor across various malignant neoplasms.

This cohort consisted exclusively of Japanese patients. Given the potential for biological treatment responses to vary in comparison with Western populations, racial disparities must be considered. According to the findings of various clinical studies, Asian patients with PCa demonstrate a high degree of responsiveness to androgen-deprivation therapy (ADT) [36,37,38]. The results of genome profiling indicated that Asian patients with PCa, especially Japanese and Chinese patients, exhibited a lower frequency of androgen receptor (AR) enhancer abnormalities and ETS gene fusions than did Western patients. In contrast, higher mutation rates have been observed in AR pathway regulators, such as FOXA1, as well as generally lower genomic instability [39]. These molecular signatures indicate a tumor phenotype that is highly dependent on the AR pathway. In fact, Japanese patients with PCa exhibit a markedly reduced risk-adjusted cancer-specific mortality rate, compared to US patients (subhazard ratio, 0.52; 95% CI, 0.40–0.68). In high-risk patients with PCa, combined ADT demonstrated superior antitumor effects compared to castration monotherapy (subhazard ratio, 0.71; 95% CI 0.56–0.91) [37]. In this study, 218 patients (44.4%) received ADT after BCR, with relatively low rates of metastatic progression (9.0%) and PCa-specific mortality (1.4%). These findings potentially indicate the biological characteristics of PCa in Japanese patients and the efficacy of ADT following BCR.

The present study had some limitations. First, this study employed retrospective data collection and lacked standardized protocols for surgical procedures and follow-ups across institutions, which potentially introduced selection and surveillance biases. The timing and regimen of salvage radiotherapy were not protocolized and were determined at the discretion of the treating physician at each institution. Second, the conventional imaging methods that were employed to diagnose metastatic progression may have resulted in an underestimation of the true incidence of metastatic disease. Additionally, variations in imaging diagnostic methods among facilities may have affected the detection sensitivity of metastatic lesions. Third, pathological evaluations were conducted at each institution without a central review, which could have resulted in interinstitutional variability in LVI and GG assessments. Patients who received treatment after the onset of BCR were included in the analysis. Fourth, data collection did not include PSA doubling time, an established prognostic factor after BCR, making it unanalyzable in this study. Finally, this analysis includes patients who received salvage therapy or adjuvant therapy, which may present a potential bias regarding the impact of differences in the interval to treatment initiation after BCR and the type of treatment on the occurrence of distant metastases.

## 5. Conclusions

In the multicenter cohort study of Japanese males with BCR after RARP, LVI was identified as an independent predictor of metastatic progression. Patients diagnosed with LVI frequently require intensive surveillance because of their elevated risk of developing distant metastases. Further studies with larger cohorts are necessary to facilitate additional risk stratification. This will optimize personalized treatment for patients undergoing RARP who have adverse pathological features such as LVI in surgical specimens.

## Figures and Tables

**Table 1:** Characteristics of patients with and without metastasis.

Characteristic	Non-Metastasis Group N = 447	Metastasis Group N = 44	*p*
Age (years, median, IQR)	68.0 (64.0, 72.0)	68.5 (61.5, 70.5)	0.584
BMI (kg/m^2^, median, IQR)	23.8 (22.1, 25.8)	24.1 (21.9, 26.0)	0.767
Initial PSA (ng/mL, median, IQR)	9.8 (6.8, 15.3)	10.6 (6.3, 15.9)	0.986
Biopsy Grade Group (number, %)			0.002
1	37 (8.3)	2 (4.5)	
2	83 (18.6)	2 (4.5)	
3	114 (25.5)	9 (20.5)	
4	147 (32.9)	14 (31.8)	
5	66 (14.8)	17 (38.6)	
Positive core rate (%, median, IQR)	33 (22, 50)	41 (25, 60)	0.105
Clinical T stage (number, %)			0.210
1	46 (10.3)	5 (11.4)	
2	316 (70.7)	26 (59.1)	
3	85 (19.0)	13 (29.5)	
NCCN risk classification (number, %)			0.009
Low	9 (2.0)	1 (2.3)	
Favorable intermediate	43 (9.6)	1 (2.3)	
Unfavorable intermediate	134 (30.0)	10 (22.7)	
High	186 (41.6)	15 (34.1)	
Very high	72 (16.1)	17 (38.6)	
Unknown	3 (0.7)	0 (0)	
Lymph node dissection (number, %)			0.862
None	77 (17.2)	9 (20.5)	
Limited	211 (47.2)	19 (43.2)	
Standard	41 (9.2)	3 (6.8)	
Extended	118 (26.4)	13 (29.5)	
Nerve-sparing (number, %)			>0.999
None	336 (75.2)	34 (77.2)	
Unilateral	92 (20.4)	9 (20.5)	
Bilateral	19 (4.33)	1 (2.3)	
Pathological grade group (number, %)			0.186
1	5 (1.1)	0 (0)	
2	95 (21.3)	4 (9.1)	
3	154 (34.5)	16 (36.4)	
4	81 (18.1)	7 (15.9)	
5	112 (25.1)	17 (38.6)	
Pathological T stage (number, %)			0.028
0	1 (0.2)	1 (2.3)	
2	190 (42.5)	12 (27.3)	
3	140 (31.3)	12 (27.3)	
4	116 (26.0)	19 (43.2)	
Lymph node positive (number, %)	61 (13.6)	7 (15.9)	0.678
Positive surgical margin (number, %)	242 (54.1)	25 (56.8)	0.733
Lymphovascular invasion (number, %)	232 (51.9)	33 (75.0)	0.003
Adjuvant therapy (number, %)			0.103
None	397 (88.8)	37 (84.1)	
Radiotherapy	17 (3.8)	0 (0)	
Hormone therapy	24 (5.4)	5 (11.4)	
Both radiotherapy and hormone therapy	9 (2.0)	2 (4.5)	
Time from RARP to BCR (months, median, IQR)	16 (5, 33)	3 (0, 16)	<0.001
Salvage radiotherapy (number, %)	181 (40.5)	12 (27.3)	0.087
Salvage hormone therapy (number, %)	194 (43.4)	24 (54.5)	0.156
Metastasis time (months, median, IQR)		24 (7, 34)	
CRPC (number, %)	7 (1.6)	19 (43.2)	<0.001
Follow-up period (months, median, IQR)	59 (43, 73)	57 (40, 71)	0.605

BMI, Body Mass Index; PSA, Prostate-Specific Antigen; NCCN, National Comprehensive Cancer Network; IQR, Interquartile range; RARP, robot-assisted radical prostatectomy; BCR, Biochemical recurrence; CRPC, Castration-Resistant Prostate Cancer.

## Data Availability

The data presented in this study are available upon request from the corresponding author due to privacy or ethical reasons.
